# Orthology for comparative genomics in the mouse genome database

**DOI:** 10.1007/s00335-015-9588-5

**Published:** 2015-07-30

**Authors:** Mary E. Dolan, Richard M. Baldarelli, Susan M. Bello, Li Ni, Monica S. McAndrews, Carol J. Bult, James A. Kadin, Joel E. Richardson, Martin Ringwald, Janan T. Eppig, Judith A. Blake

**Affiliations:** The Jackson Laboratory, Bar Harbor, ME 04609 USA

## Abstract

The mouse genome database (MGD) is the model organism database component of the mouse genome informatics system at The Jackson Laboratory. MGD is the international data resource for the laboratory mouse and facilitates the use of mice in the study of human health and disease. Since its beginnings, MGD has included comparative genomics data with a particular focus on human–mouse orthology, an essential component of the use of mouse as a model organism. Over the past 25 years, novel algorithms and addition of orthologs from other model organisms have enriched comparative genomics in MGD data, extending the use of orthology data to support the laboratory mouse as a model of human biology. Here, we describe current comparative data in MGD and review the history and refinement of orthology representation in this resource.

## Introduction

The fundamental mission of the mouse genome database (MGD) is to facilitate the use of the laboratory mouse as a model system for understanding human biology and disease. The mouse is the premier model organism: it is a mammalian system in which all life stages can be accessed, for which many phenotypically well-characterized inbred strains exist, for which a completely sequenced reference genome is publicly available, and for which many genomic tools for comparative and experimental manipulation have been developed.

Orthology is a key to the use of mouse as a model for human biology. Since genes that share close evolutionary relationships are likely to function in similar ways, many applications leverage phylogenetic relationships to propagate inferred functional annotation among related genes. As a model organism database, MGD has long exploited orthologous mammalian relationships to relate data between human and mouse and to infer gene function from experimentally studied orthologs. As a closely related species, studies in the rat can also provide a great deal of experimental data that are complementary to that of the mouse when inferred via orthology. Further orthology assertions in other well-studied vertebrates and to more distantly related organisms such as fruitfly and yeast can be informative as well. In practice, the important distinction of experimentally determined function and inferred function is always recorded, along with the source from which function is inferred.

Until recently, cross-species comparison in MGD permitted only a single gene per species in an orthology set. However, now with the more comprehensive annotation of genomes using a variety of algorithms, MGD has moved to accommodate incorporation of gene sets where there may be more than one gene per species in the orthology set. We implemented this many-to-many homology paradigm in 2013 to better reflect current understanding of the complexity of the relationships among the genes of mouse, human, and rat. Although one-to-one orthology assertions between mouse and human or rat genes still hold for most protein-coding genes, MGD can now more clearly represent complex cases in which one species has multiple genes in the same homology class due to paralogous gene duplication.

Semantically, the terms ‘homology’ and ‘orthology’ are often used interchangeably and sometimes inconsistently. Here, we use the term ‘homology’ to include both ‘orthology’—the phylogenetic relationship between genes in different species that results from a speciation event—and ‘paralogy’—the relationship among genes in the same species that results from a gene duplication event within the species. When discussing MGD’s one-to-one paradigm and, specifically the relationship of mouse and human genes, here we use the term ‘orthology.’ When discussing MGD’s newer many-to-many paradigm, which may include the relationship among one or more mouse and one or more human genes, we will use the term ‘homology.’

As a result of the change to a many-to-many homology paradigm and with the inclusion of phylogeny-based function predictions, MGD has improved the availability of homology rule-based annotations and relationships to provide robust interconnections between mouse and human genetic and genomic data.

## Historical perspective

MGD was first released on the World Wide Web in June of 1994. It was the consolidation of other separate databases at The Jackson Laboratory, many of which included some statement of human and mouse gene orthology particularly in the context of comparative mapping data. MGD, from the start, was the authority for mouse nomenclature, for the annotation and characterization of the mouse genome, and for the representation of mouse–human orthology. Early efforts emphasized working in collaboration with human genome annotation streams to remove redundancy and to deploy controlled vocabularies and syntax. Orthology data in MGD were exclusively based on literature describing experimental analysis and requiring citation support (Blake et al. 1997). In 2004, MGD assertions were augmented with automated assertions from HomoloGene (Table 1) (NCBI Resource Coordinators 2014). From 2004 until 2013, the group of mammalian species for which orthology data were collected included selected primates, rodents deemed most relevant due to experimental status, and several domestic species. In coordination with such assertions, graphical comparative maps provided a detailed chromosomal view of conserved segments between mouse and other mammalian species.

**Table 1:** A chronological list of significant changes to MGD orthology representation

Year	Significant changes to MGD orthology representation
1994	MGD went online
1997	Determination of homology in MGD is based on experimental analysis
	Interactive Oxford Grids displaying comparative mapping between two species are available for mouse, human, rat, cow, pig, sheep, and cat
1998	Over 2500 mouse/human homologies are found in MGD as well as a more limited number of homology assertions for >60 other mammalian species
	Mammalian homologs can also be displayed as part of the detail for graphical map displays
2000	The type of evidence used to determine the homology relationship is provided: Sequence similarity, conserved location, or functional analysis
	MGD starts to emphasize the relationship of mouse genes to those in other model organisms such as Drosophila
2002	MGD provides gene family pages that summarize information about curated orthology assertions of mouse, human, and rat orthologs
2004	MGD works with the HomoloGene resource at the NCBI to reciprocally incorporate some of the HomoloGene computational three-way reciprocal best-hit sets into the MGI system
2005	MGD’s priority effort focuses on the creation of orthology sets among mouse, human, and rat
2007	MGD incorporates UniProt Protein Information Resource Superfamily (PIRSF) protein classifications into a Protein Superfamily Vocabulary Browser
	MGD provides new mouse–human–rat comparative GO graphs
2008	MGD includes links to the TreeFam resource
2013	A banner displaying information about the human ortholog of each mouse gene is added to the Gene Detail pages in MGD to improve comparisons of gene–disease associations in mouse and human
	MGI implements a many-to-many homology paradigm to better reflect current understanding about the relationships between genes among mammals
2015	MGI expands the many-to-many homology paradigm to include HGNC orthology assertions to maximize the use of human:mouse comparative genomics

Related to the representation of orthology, MGD has a long history of incorporating data on phylogenetically related gene families. Since 2002, MGD has provided gene family pages that summarize information about mouse, human, and rat orthologs. These curated representations of gene families incorporated the combined evaluations of mouse, human, and rat scientific curators with the input of the scientific research community to evaluate and clarify the gene family relationships (Blake et al. 2002). In 2007 (Eppig et al. 2007), MGD incorporated UniProt PIRSF (Protein Information Resource Superfamily) (Wu et al. 2004) protein classifications into a Protein Superfamily Vocabulary Browser, links to VISTA homology plots (Frazer et al. 2004), and links to TreeFam (Li et al. 2006), which provided curated information about ortholog and paralog assignments and the evolutionary history of various gene families. In 2011, MGD replaced the TreeFam resource with Ensembl Compara Gene Tree (Cunningham et al. 2015).

The advent of whole-genome sequencing from several of the Human Genome Project model organisms, coupled with the import of HomoloGene data, greatly expanded the representation of orthologies in MGD. The one-to-one orthology paradigm core to MGD orthology curation together with the import of genome-wide HomoloGene data (still restricting the orthology sets to one mouse gene to one human or rat gene cases) increased mouse-containing orthology pairs from about 2500 mouse–human pairs in 1998, to 9987 mouse–human pairs in 2004, to 17,852 in 2011, and to 17,773 mouse–human and 17,253 mouse–rat orthology pairs in 2013 (Table 2). With the inclusion of many-to-many homology sets from HomoloGene (described below), the counts adjusted somewhat to fewer mouse–human homologs (genes associated via homology sets) and more mouse–rat homologs (January 2015 data: 17,055 mouse–human and 18,461 mouse–rat).

**Table 2:** A summary of the increased representation of mouse:human and mouse:rat orthology sets in MGD

	Mouse/human orthologs	Mouse/rat orthologs
1998	2500	
2002	6123	
2003	7488	
2004	9987	
2005	14,893	
2006	15,849	15,532
2007	15,672	14,758
2008	16,927	15,801
2009	16,685	15,787
2010	17,787	16,768
2011	17,852	
2012	17,847	16,686
2013	17,773	17,253
2014	17,092	17,811
2015	17,055	18,461

## Recent changes in homology representation in MGD

In 2013, we implemented a many-to-many homology paradigm to better reflect current understanding of the complexity of the relationships among the genes of mouse, human, and rat (Blake et al. 2014). This change involved moving from MGD-vetted one-to-one orthology assertions to the comprehensive use of HomoloGene as its homology authority. Along with this change, we extended the representation of homology sets from mammalian-only to vertebrate-inclusive sets, thus now representing and utilizing HomoloGene homology assertions from such well-studied species as zebrafish (*Danio rerio*) and chicken (*Gallus gallus*).

Although MGD had been incorporating orthology data from the NCBI HomoloGene resource for some years, these data were restricted to the one-to-one cases of orthology among mammals. This restriction included more than 90 % of protein-coding genes, but now MGD can more clearly represent loci that include a more complex sequence of speciation and gene duplication or deletion events. For example, MGI can now more clearly represent complex relationships in cases such as *Serpina1a* (MGI:891971), where phylogenetic analysis shows five mouse genes and one human gene in the same homology class as a result of paralogous gene duplication events in the mouse.

Although most homology assertions resulting from different algorithms agree, there are differences that have implications in the assessment of mouse models and their relationships to human diseases. In order to maximize the use of human–mouse homology sets for comparative genomics, the May 2015 release of MGI introduced the use of HUGO Gene Nomenclature Committee (HGNC) (Gray et al. 2015) as a second external homology source to complement HomoloGene for mouse–human homology. We developed rules that merge and select human–mouse homology assertions from HomoloGene and HGNC data sets; these are discussed below. This most recent MGI release also includes links from the mouse gene detail page to all associated (via both HomoloGene and HGNC) human gene homology sets defined by a variety of sequence-based and phylogeny-based algorithms represented in the HGNC resource, Human Comparative Orthology Prediction (HCOP) (Eyre et al. 2007).

## Representing homology in MGD

The representation of homology does not exist in isolation at MGD. It depends on the development of an unambiguous catalog of mouse genes; it is simplified by a common standardized gene naming system; and it is facilitated by the use of standard vocabularies to describe functional and phenotypic attributes.

### Unified mouse gene catalog

The catalog of mouse genes in MGD serves as the foundation for functional annotation of all genes and genome features in the database. The MGD gene curation process integrates gene predictions from Ensembl, NCBI, and Vega (Wilming et al. 2008) into a single, non-redundant catalog (Zhu et al. 2015). The unified gene catalog is updated when new gene predictions are released. The concept of gene in the unified mouse gene catalog refers to the computational prediction of structural genome features including protein-coding and non-protein-coding genes. This allows researchers to obtain a comprehensive list of mouse genes from a single source.

### Mouse nomenclature

The curation of a unique set of symbols and names for mouse genes facilitates the integration of genetic and genomic data. The Mouse Genomic Nomenclature Committee assigns unique symbols and names to mouse genes under the guidelines set by the International Committee on Standardized Genetic Nomenclature for Mice (http://www.informatics.jax.org/mgihome/nomen/inc.shtml) working with nomenclature specialists for human [HUGO Gene Nomenclature Committee (http://www.genenames.org/)] and rat [Rat Genome Nomenclature Committee (http://rgd.mcw.edu/nomen/nomen.shtml) at the Rat Genome Database (RGD) (Shimoyama et al. 2015)], to provide consistent nomenclature for mammalian species. MGD initiated the merging of mouse and rat gene, allele, and strain nomenclature guidelines via the International Committee for Standardized Nomenclature in Mice and the Rat Genome Nomenclature Committee in 2003. Now, there is a common standard for nomenclature in rodent species that provides a simplified system for researchers and that should lessen the ambiguity of species-specific names in mice and rats and encourage the co-naming of gene orthologs.

### Controlled vocabularies

**Fig. 1:**
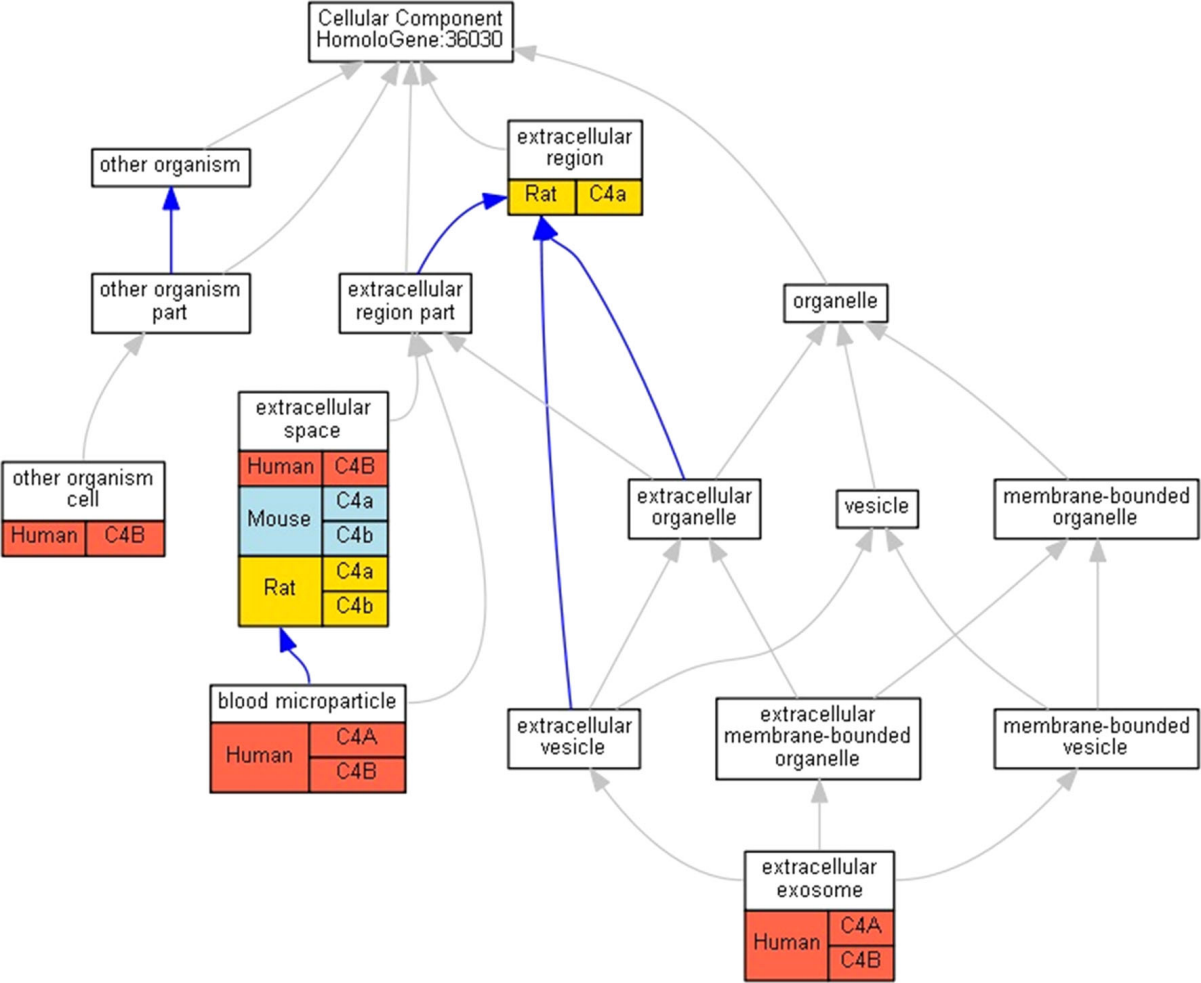
Comparative graphs present human, mouse, and rat GO annotations in the context of the ontology structure to better enable comparison among organisms. The graphs have been adapted, as shown here, to accommodate MGI’s many:many homology paradigm

Standardization of terms and vocabularies within MGD facilitates data entry and searching. Standardized classification terms in the unified mouse gene catalog are provided for genome feature type (e.g., protein-coding gene, pseudogene, and noncoding RNA). Since 2006, to enhance the representation of relationships between mouse models and human diseases, MGD curators have associated mouse mutant genotypes with disease terms from Online Mendelian Inheritance in Man (OMIM 2015), a text-based compendium of human genes and diseases maintained by the Johns Hopkins University (http://www.ncbi.nlm.nih.gov/omim/). OMIM disease terms are available at MGD as a vocabulary to allow users to access these data from a human-centric as well as a mouse-centric view. MGD makes use of several biomedical ontologies for gene annotation: gene product function data are annotated using the Gene Ontology (Ashburner et al. 2000; Gene Ontology Consortium 2015); mouse phenotype data using the Mammalian Phenotype Ontology (MP) (Smith et al. 2005); and expression data using the ontology of mouse developmental anatomy (EMAP/EMAPA) (Hayamizu et al. 2013). These efforts to incorporate standard vocabularies enable data exchange, retrieval, and integration at MGD. For example, we use homology sets to produce comparative graphs that present human, mouse, and rat GO experimental annotations in the context of the ontology structure to better enable comparison among these organisms (see Fig. 1).

## Use of orthology to infer gene function

Orthology is a key aspect of incorporating data supporting inferred functioning of mouse genes. MGD is one of the founding groups of the gene ontology (GO) and continues to be a core group in the Gene Ontology Consortium (GOC). MGD is the authoritative source of GO annotations for mouse genes (Eppig et al. 2015) using the GOC standards for data curation and integration. MGD also has in place an automated pipeline that brings experimentally based annotations into the MGD system from other model organism systems such as from the RGD utilizing the orthology assertions generated from the MGD orthology pipeline (Drabkin et al. 2015). In addition, MGD loads functional annotations derived from the GO phylogenetic annotation process (Gaudet et al. 2011) by which experimental annotations are overlaid on PANTHER gene family trees and inferential annotations are applied based on phylogenetic subclassifications.

## Maximizing use of orthology sets for human/mouse comparative genomics

A primary use of orthology data in MGD is to show human–mouse disease phenotype concordance, potential concordant models of human disease, and potential candidate human disease genes based on non-concordant models. The many-to-many homolog paradigm has enriched the perspective of concordance in MGD, as shared disease phenotypes among paralogs within a mouse–human homology cluster can now be realized. HomoloGene data have the advantage of being genome wide, yet the HomoloGene methodology’s reliance on sequence similarity occasionally produces orthology clusters that conflict with clusters based on phylogenetic trees or curated orthology assertions. For example, HomoloGene groups the human glycerol kinase (*GK*) gene with the mouse glycerol kinase-like 1 gene (*Gykl1*) (HomoloGene: 21848). HGNC groups human *GK* with the mouse glycerol kinase gene (*Gk*). Both the human *GK* and mouse *Gk* genes are associated with the inherited disease Glycerol Kinase Deficiency (OMIM 307030). The HomoloGene view fails to reflect this concordance, the HGNC view does.

**Fig. 2:**
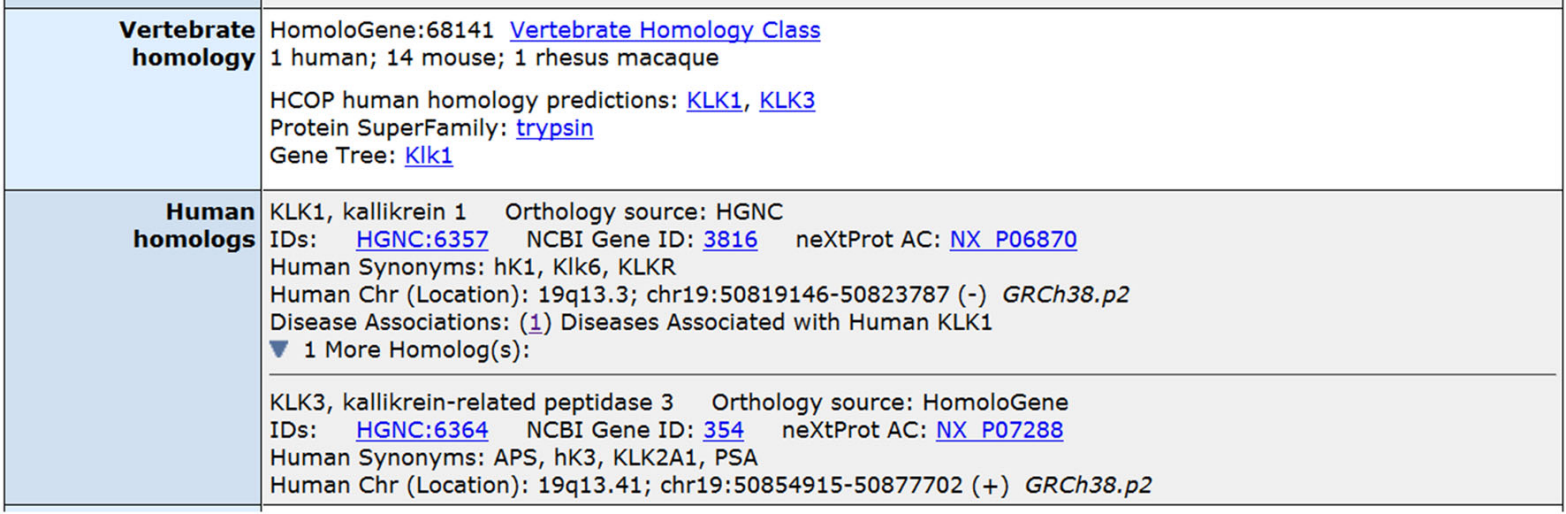
The Vertebrate homology ribbon on the mouse gene *Klk1* detail page displays information on the HomoloGene class that contains 1 human and 14 mouse genes. There are links to HCOP homology predictions for the human gene *KLK3* called by HomoloGene and to *KLK1* called by HGNC. The Human homolog ribbon displays additional information on both human genes associated with *Klk1*. The orthology data presented on the gene detail page are inclusive of orthologs called by both sources

**Fig. 3:**
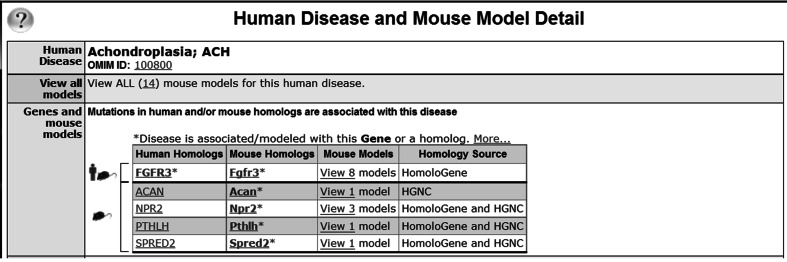
The Human Disease and Mouse Model Detail page provides a direct comparison of mouse and human orthologs of genes associated with a human disease. These associations are based on the hybrid homology rules. In case where HomoloGene and HGNC agree, as in the last three genes displayed here, that agreement is shown in the last column. In cases where the orthology sets disagree, our rules select the more inclusive set; as shown here the HomoloGene pair *FGFR3*–*Fgfr3* and the HGNC set for *ACAN*–*Acan*. The hybrid homology set includes 25,999 ortholog sets from HomoloGene and 33,717 from HGNC

It is for cases like the above-mentioned glycerol kinase that MGD has extended its representation of mouse–human homology from only using HomoloGene to including HGNC assertions. A complication however with homology data from HGNC and other gene-centric resources is that the data are available only as pair-wise homology assertions, as opposed to accessioned homology clusters as is the case with data from a dedicated homology resource such as HomoloGene. To load HGNC homology data, we compute homology clusters from pair-wise input data, allowing clusters to contain one or more human genes and zero, one, or more mouse genes per cluster. MGD now incorporates homology clusters from both HomoloGene and HGNC, benefiting from the strengths of each resource. Both perspectives are displayed on the MGD gene detail page Vertebrate homology and Human homolog sections (see Fig. 2) and on the Human Disease and Mouse Model Detail pages (see Fig. 3), and are incorporated into gene nomenclature searches. The details of the homology clusters from HomoloGene or HGNC can be viewed on the MGD Homology Detail pages (which can be accessed using the “Vertebrate Homology Class” link of the gene detail page, see Fig. 2).

Homology conflicts between HomoloGene and HGNC can complicate interpretation of potential human–mouse concordance. To avoid this complication, only one homology perspective is used (HomoloGene or HGNC) on MGD pages where homology is displayed (or searched) in the context of phenotypes or disease. These pages include the Human–Mouse: Disease Connection (HMDC, www.diseasemodels.org) and Disease Detail pages. HMDC is a translational tool, introduced to MGD in 2014, that allows users to explore gene–phenotype–disease relationships between human and mouse. To select a single homology perspective, we first compute composites (connected components) of HomoloGene and HGNC clusters. These are sets of HomoloGene and HGNC clusters that share at least one mouse or human gene in common. We then apply a set of rules to select either the HomoloGene or HGNC cluster(s) from each connected component and store the selected cluster(s) as a separate, “Hybrid” homology cluster set. The Hybrid clusters retain the source of the original clusters selected (HomoloGene, HGNC, or both if the clusters from both sources are identical). The rules to select clusters for the Hybrid set are designed to optimize mouse–human connections for disease and phenotype displays. For cases in which HomoloGene and HGNC disagree on clustering, the cluster with mouse and human genes has precedence for selection over the cluster without mouse. For cases in which both HomoloGene and HGNC have mouse–human clusters, the HGNC cluster is selected since it is deemed more robust for mouse–human homology. Note that this selection is only for searching in the context of disease and phenotypes; both HomoloGene and HGNC assertions are displayed on the mouse gene detail pages. For example, for connected components that contain complex conflicts, we select HGNC’s representation. Thus, a search for “Glycerol Kinase Deficiency” on the HMDC grid will return the human *GK* gene paired only with mouse *Gk*, and the disease association will be shown for the human and mouse genes. The following sections describe how these rules are applied when searching for orthology-based data in MGD.

## Searching for mouse models of human disease using the Hybrid Homology

Entering a disease term (OMIM) or a Mammalian Phenotype term (MP) in the MGD Quick Search Tool returns mouse genes and alleles associated with the disease or phenotype entered. For disease searches, this association between a mouse gene and the disease can be due to either a mutation in the mouse gene that models the disease or the orthology between the mouse gene and a human gene associated with the disease. Mutations in mouse orthologs of human disease genes represent potential concordant disease models. When MGD uses human–mouse orthology to return mouse genes from a disease or phenotype search, the Hybrid Homology is used.

**Fig. 4:**
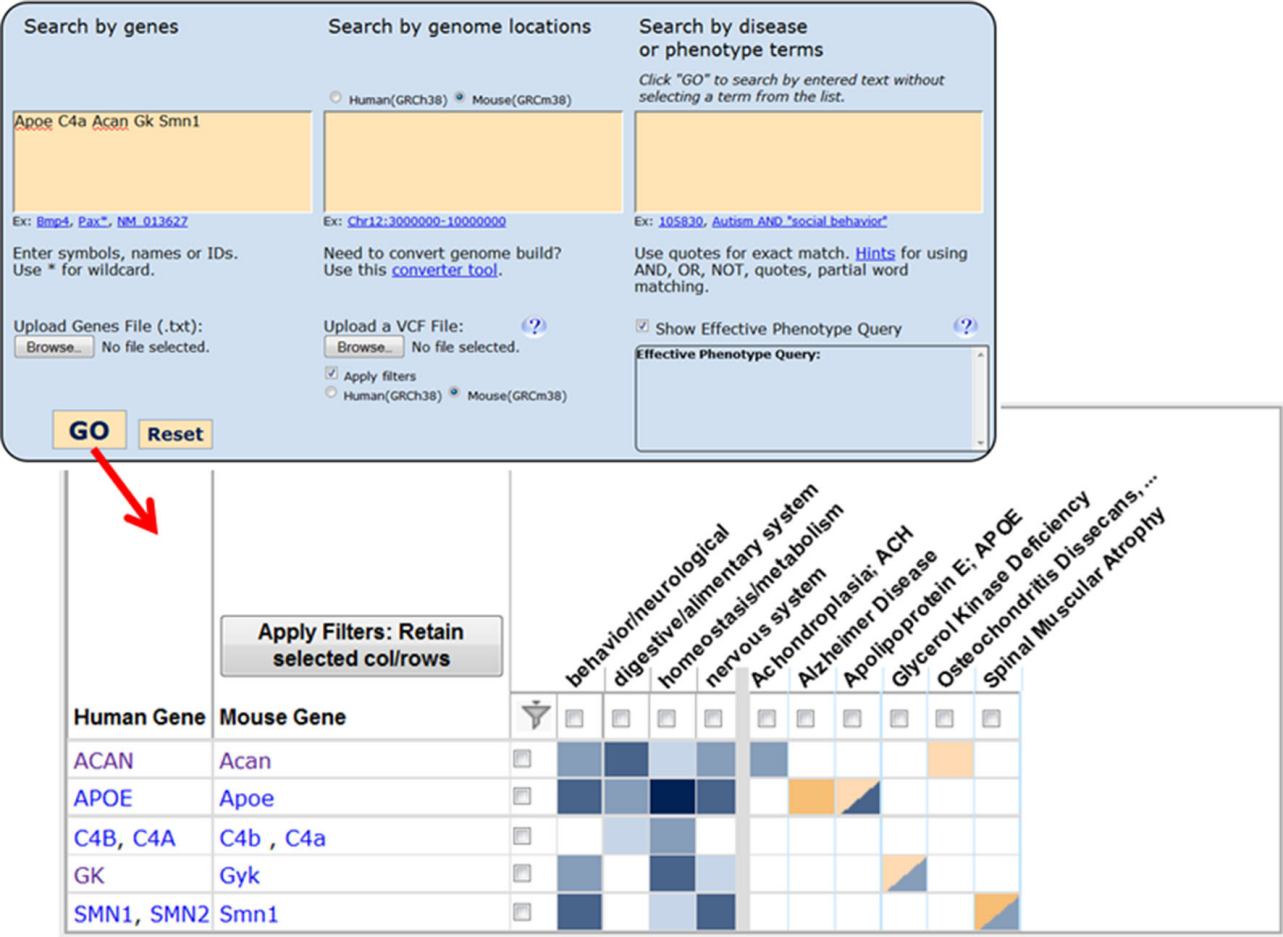
Searching the HMDC with mouse or human symbols returns a row with the Hybrid homology set for each gene matching a search term. The mouse phenotype annotations and human and mouse disease annotations for genes in the homology set are shown in the row. The matrix shown in the figure has been filtered to reduce the number of rows and columns. The source for each homology cluster is: *ACAN, Acan, HGNC; APOE, Apoe*, HomoloGene, and HGNC; *C4A, C4B, C4a, C4b*, HomoloGene, and HGNC; *GK, Gk, HGNC; *and *SMN1, SMN2, Smn1*, HomoloGene. The *C4A, C4B, C4a, C4b* represents a case where MGI constructed a multi-gene homology cluster from several HGNC pairs. This constructed cluster is identical to the one in HomoloGene. For *ACAN, Acan* and *SMN1, SMN2, Smn1* clusters, the source selected is the only one that had a cluster containing both mouse and human genes. For *GK, Gk* both sources had clusters containing mouse and human genes, the hybrid uses HGNC clusters in these cases

Similarly, disease or mouse phenotype searches in the HMDC return mouse and human genes associated with the term entered. The HMDC constructs a grid in which rows are homolog clusters containing human and/or mouse genes returned and columns are phenotypes for the mouse gene(s) and diseases for the human and mouse genes returned (see Fig. 4). The homolog cluster display on the HMDC grid makes the ‘Connection’ between mouse phenotypes, human diseases, and their associated genes. The homolog clusters shown on the HMDC grid and used in phenotype/disease searches are Hybrid clusters.

## Links to mouse ortholog gene expression using resource-specific orthology assertions

MGD is a core component of the larger MGI system. Another part of MGI, the Gene Expression Database (GXD), integrates curated mouse gene expression data, placing the diverse biological data types in MGI in a searchable expression context (Smith et al. 2015). MGI gene detail pages feature a GXD section that offers summary-level views of expression data for the mouse gene and links to GXD pages that provide more detailed views of primary expression data. Included in the GXD section are links to external expression data for vertebrate homologs of the mouse gene, including chicken (GEISHA) (Bell et al. 2004; Darnell et al. 2007), frog (Xenbase) (Karpinka et al. 2015), and zebrafish (ZFIN) (Bradford et al. 2011). These links to expression in vertebrate model organisms rely on coordinated orthology between MGI and the model organism resources, as each resource asserts homology to mouse genes. HomoloGene includes these model organisms, but discrepancies exist between HomoloGene’s view and the homology assertions from these resources. To ensure accurate links to these external resources, MGI loads homology data from each, where expression data for the vertebrate homologs are available, taking full advantage of an infrastructure designed to accommodate homology cluster data from multiple sources. As with data from HGNC, homology data from these other vertebrate resources is pair wise, and thus MGI computes homology clusters for each resource. No Hybrid homology clusters (between HomoloGene and these resources) are necessary, since the homology in this case is used to direct specific links back to these resources. These links extend expression context to a valuable comparative genomics perspective.

## Summary

The MGD resource integrates genetic and genomic data relevant to the laboratory mouse with the core objective of facilitating the use of the mouse as a model of human biology. Key to this work is comprehensive and detailed representation of the homologous relationship between mouse genes and the genes of other organisms, in particular, human genes. The changes we have implemented in homology representation over time, including paralogy and multiple (often complementary) homology resources, have helped improve the use of MGD as a resource for comparative genomics by expanding the view of potential mouse models for human disease. The many-to-many homolog paradigm has enriched the perspective of concordance in MGD, as shared disease phenotypes among paralogs within a mouse–human homology cluster can now be realized. MGD is also actively exploring the incorporation of phylogenetic tree-based orthology predictions, such as implementing the load of the PANTHER gene families (http://www.pantherdb.org/genes/), in the near future. As our understanding of human and mouse genetic and genomic features and their relationships to each other continues to emerge, MGD will continue to refine our representation and utilization of this knowledge as a core component of our work.
